# *catena*-Poly[[(dichloridozinc)-μ-4,4′-bis­(1*H*-imidazol-1-yl)biphenyl-κ^2^ *N* ^3^:*N* ^3′^] 0.25-hydrate]

**DOI:** 10.1107/S1600536812009543

**Published:** 2012-03-14

**Authors:** Yong-Xia Ning, Wen Fan, Gang Xie

**Affiliations:** a Key Laboratory of Synthetic and Natural Functional Molecule Chemistry (Ministry of Education), College of Chemistry & Materials Science, Northwest University, Xi’an 710069, People’s Republic of China

## Abstract

In the title one-dimensional coordination polymer, {[ZnCl_2_(C_18_H_14_N_4_)]·0.25H_2_O}_*n*_, the Zn^II^ atom is coordinated by two chloride ions and two 4,4′-bis­(1*H*-imidazol-1-yl)biphenyl ligands, generating a distorted tetra­hedral ZnCl_2_N_2_ geometry. The dihedral angle between the benzene rings of the ligand is 51.0 (1)° and the dihedral angles between the benzene rings and their attached imidazole rings are 18.7 (2) and 45.9 (1)°. The bridging ligand leads to [10-1] polymeric chains in the crystal and the disordered water mol­ecule (occupancy 0.25) forms O—H⋯Cl hydrogen bonds.

## Related literature

For background to coordination polymers containing imidazole-derived ligands, see: Li *et al.* (2010 ▶, 2011 ▶).
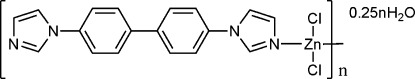


## Experimental

### 

#### Crystal data

[ZnCl_2_(C_18_H_14_N_4_)]·0.25H_2_O
*M* *_r_* = 427.11
Monoclinic, 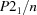

*a* = 8.1565 (16) Å
*b* = 12.554 (3) Å
*c* = 18.411 (4) Å
β = 101.08 (3)°
*V* = 1850.0 (6) Å^3^
*Z* = 4
Mo *K*α radiation
μ = 1.63 mm^−1^
*T* = 293 K
0.25 × 0.22 × 0.20 mm

#### Data collection

Rigaku Mercury CCD diffractometer
Absorption correction: multi-scan (*CrystalClear*; Rigaku/MSC, 2005 ▶) *T*_min_ = 0.687, *T*_max_ = 0.737
15655 measured reflections
3256 independent reflections
2622 reflections with *I* > 2σ(*I*)
*R*_int_ = 0.058

#### Refinement

*R*[*F* ^2^ > 2σ(*F* ^2^)] = 0.052
*wR*(*F* ^2^) = 0.092
*S* = 1.16
3256 reflections
230 parameters
H-atom parameters constrained
Δρ_max_ = 0.35 e Å^−3^
Δρ_min_ = −0.31 e Å^−3^

### 

Data collection: *CrystalClear* (Rigaku/MSC, 2005 ▶); cell refinement: *CrystalClear*; data reduction: *CrystalClear*; program(s) used to solve structure: *SHELXS97* (Sheldrick, 2008 ▶); program(s) used to refine structure: *SHELXL97* (Sheldrick, 2008 ▶); molecular graphics: *SHELXTL* (Sheldrick, 2008 ▶); software used to prepare material for publication: *SHELXTL*.

## Supplementary Material

Crystal structure: contains datablock(s) I, global. DOI: 10.1107/S1600536812009543/hb6628sup1.cif

Structure factors: contains datablock(s) I. DOI: 10.1107/S1600536812009543/hb6628Isup2.hkl

Additional supplementary materials:  crystallographic information; 3D view; checkCIF report

## Figures and Tables

**Table 1:** Selected bond lengths (Å)

Zn1—N1	2.021 (3)
Zn1—N3^i^	2.024 (3)
Zn1—Cl2	2.2368 (13)
Zn1—Cl1	2.2370 (12)

Symmetry code: (i) 
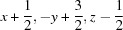
.

**Table 2:** Hydrogen-bond geometry (Å, °)

*D*—H⋯*A*	*D*—H	H⋯*A*	*D*⋯*A*	*D*—H⋯*A*
O1*W*—H1*A*⋯Cl2^ii^	0.85	2.55	3.270 (14)	143
O1*W*—H1*B*⋯Cl1^iii^	0.85	2.19	3.038 (14)	179

Symmetry codes: (ii) 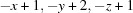; (iii) 
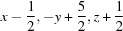
.

**Fig. 1.**
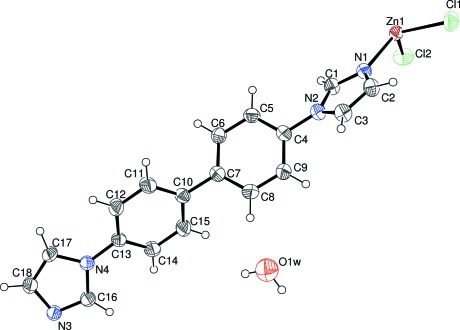
The molecular structure of (I). Displacement ellipsoids are drawn at the 30% probability level.

**Fig. 2.**
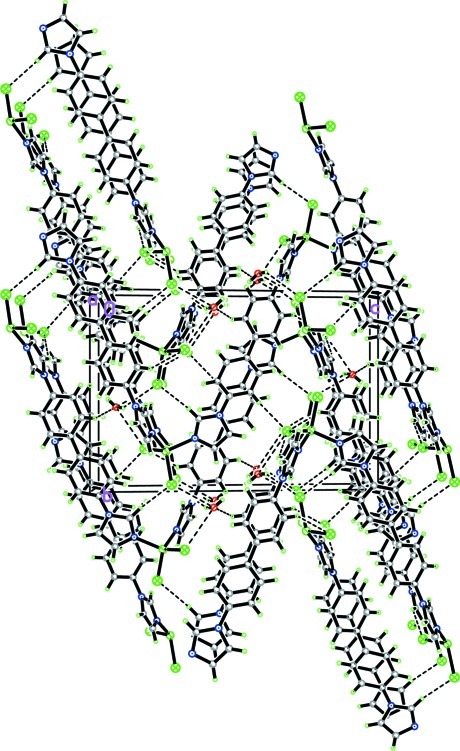
The crystal packing for (I).

## supplementary crystallographic information

### Comment

In recent years, imidazole has been well used in crystal engineering, and a large number of imidazole-containing flexible ligands have been extensively studied. However, to our knowledge, the research on imidazole ligands bearing rigid spacers is less developed (Li *et al.*, 2010; Li *et al.*, 2011).

Single-crystal X-ray diffraction analysis reveals that the title compound (I) crystallizes in the monoclinic space group P21/n. For the title compound, the geometry of the Zn^II^ ion is bound by two imidazole rings of individual L ligands, and two chlorine anions, which illustrates a slightly distorted tetrahedral coordination environment (Fig 1). Notably, as shown in Fig. 2, the four-coordinate Zn^II^ center is bridged by the ligand L to form an infinite one-dimensional architecture.

### Experimental

A mixture of CH_3_OH and H_2_O (1:1, 8 ml), as a buffer layer, was carefully layered over a solution of ZnCl_2_ (0.02 mmol) in H_2_O (6 ml). Then a solution of 4,4'-bis(1*H*-imidazol-1-yl)phenyl (L, 0.06 mmol) in CH_3_OH (6 ml) was layered over the buffer layer, and the resultant reaction was left to stand at room temperature. After *ca* three weeks, colorless block single crystals appeared at the boundary. Yield: ~25% (based on L).

### Refinement

The dispalcement parameters for the water O atom were very large at full occupancy. When refined, its fractional occupancy converged to close to 0.25 and was then set at this value. C-bound H atoms were positioned geometrically and refined in the riding-model approximation, with C—H = 0.93Å and *U*_iso_(H) = 1.2*U*_eq_ (C).

### Crystal data

**Table:**

[ZnCl_2_(C_18_H_14_N_4_)]·0.25H_2_O	*F*(000) = 866
*M**_r_* = 427.11	*D*_x_ = 1.533 Mg m^−^^3^
Monoclinic, *P*2_1_/*n*	Mo *K*α radiation, λ = 0.71073 Å
*a* = 8.1565 (16) Å	Cell parameters from 15648 reflections
*b* = 12.554 (3) Å	θ = 3.0–27.6°
*c* = 18.411 (4) Å	µ = 1.63 mm^−^^1^
β = 101.08 (3)°	*T* = 293 K
*V* = 1850.0 (6) Å^3^	Block, colorless
*Z* = 4	0.25 × 0.22 × 0.20 mm

### Data collection

**Table:**

Rigaku Mercury CCD diffractometer	3256 independent reflections
Radiation source: fine-focus sealed tube	2622 reflections with *I* > 2σ(*I*)
Graphite monochromator	*R*_int_ = 0.058
Detector resolution: 9 pixels mm^-1^	θ_max_ = 25.0°, θ_min_ = 3.1°
ω scans	*h* = −9→9
Absorption correction: multi-scan (*CrystalClear*; Rigaku/MSC, 2005)	*k* = −14→14
*T*_min_ = 0.687, *T*_max_ = 0.737	*l* = −21→21
15655 measured reflections	

### Refinement

**Table:**

Refinement on *F*^2^	Primary atom site location: structure-invariant direct methods
Least-squares matrix: full	Secondary atom site location: difference Fourier map
*R*[*F*^2^ > 2σ(*F*^2^)] = 0.052	Hydrogen site location: inferred from neighbouring sites
*wR*(*F*^2^) = 0.092	H-atom parameters constrained
*S* = 1.16	*w* = 1/[σ^2^(*F*_o_^2^) + (0.0291*P*)^2^ + 1.2964*P*] where *P* = (*F*_o_^2^ + 2*F*_c_^2^)/3
3256 reflections	(Δ/σ)_max_ = 0.001
230 parameters	Δρ_max_ = 0.35 e Å^−^^3^
0 restraints	Δρ_min_ = −0.31 e Å^−^^3^

### Special details

**Table:**

Geometry. All e.s.d.'s (except the e.s.d. in the dihedral angle between two l.s. planes) are estimated using the full covariance matrix. The cell e.s.d.'s are taken into account individually in the estimation of e.s.d.'s in distances, angles and torsion angles; correlations between e.s.d.'s in cell parameters are only used when they are defined by crystal symmetry. An approximate (isotropic) treatment of cell e.s.d.'s is used for estimating e.s.d.'s involving l.s. planes.
Refinement. Refinement of *F*^2^ against ALL reflections. The weighted *R*-factor *wR* and goodness of fit *S* are based on *F*^2^, conventional *R*-factors *R* are based on *F*, with *F* set to zero for negative *F*^2^. The threshold expression of *F*^2^ > σ(*F*^2^) is used only for calculating *R*-factors(gt) *etc*. and is not relevant to the choice of reflections for refinement. *R*-factors based on *F*^2^ are statistically about twice as large as those based on *F*, and *R*- factors based on ALL data will be even larger.

### Fractional atomic coordinates and isotropic or equivalent isotropic displacement parameters (Å^2^)

**Table:**

	*x*	*y*	*z*	*U*_iso_*/*U*_eq_	Occ. (<1)
N3	0.1785 (4)	0.2607 (2)	0.64336 (16)	0.0383 (8)	
C16	0.2231 (5)	0.3586 (3)	0.6299 (2)	0.0405 (10)	
H16	0.2570	0.4097	0.6663	0.049*	
N4	0.2135 (4)	0.3755 (2)	0.55687 (16)	0.0380 (8)	
C13	0.2497 (5)	0.4730 (3)	0.5231 (2)	0.0370 (9)	
C14	0.3472 (6)	0.5486 (3)	0.5650 (2)	0.0570 (12)	
H14	0.3939	0.5347	0.6143	0.068*	
C15	0.3752 (6)	0.6452 (3)	0.5337 (2)	0.0566 (12)	
H15	0.4387	0.6964	0.5630	0.068*	
C10	0.3122 (5)	0.6681 (3)	0.4604 (2)	0.0385 (9)	
Zn1	0.67056 (5)	1.30302 (3)	0.24360 (2)	0.03635 (15)	
N1	0.5135 (4)	1.2116 (2)	0.28937 (16)	0.0371 (8)	
N2	0.4081 (4)	1.0778 (2)	0.34273 (17)	0.0385 (8)	
C1	0.5385 (5)	1.1127 (3)	0.3145 (2)	0.0411 (10)	
H1	0.6337	1.0728	0.3128	0.049*	
C2	0.3587 (5)	1.2400 (3)	0.3021 (2)	0.0430 (10)	
H2	0.3073	1.3055	0.2900	0.052*	
C3	0.2931 (5)	1.1594 (3)	0.3345 (2)	0.0448 (10)	
H3	0.1897	1.1585	0.3488	0.054*	
C4	0.3885 (4)	0.9745 (3)	0.3733 (2)	0.0361 (9)	
C5	0.4253 (5)	0.8852 (3)	0.3368 (2)	0.0429 (10)	
H5	0.4660	0.8918	0.2931	0.051*	
C6	0.4018 (5)	0.7850 (3)	0.3650 (2)	0.0413 (10)	
H6	0.4273	0.7245	0.3402	0.050*	
C7	0.3404 (5)	0.7745 (3)	0.4297 (2)	0.0384 (9)	
C8	0.3060 (5)	0.8666 (3)	0.4660 (2)	0.0481 (11)	
H8	0.2670	0.8609	0.5101	0.058*	
C9	0.3285 (5)	0.9661 (3)	0.4380 (2)	0.0471 (10)	
H9	0.3033	1.0269	0.4625	0.056*	
C18	0.1364 (6)	0.2126 (3)	0.5758 (2)	0.0503 (11)	
H18	0.0996	0.1427	0.5680	0.060*	
C17	0.1562 (6)	0.2820 (3)	0.5223 (2)	0.0520 (12)	
H17	0.1352	0.2690	0.4716	0.062*	
C12	0.1883 (5)	0.4928 (3)	0.4495 (2)	0.0505 (11)	
H12	0.1239	0.4417	0.4205	0.061*	
C11	0.2221 (5)	0.5887 (3)	0.4187 (2)	0.0503 (11)	
H11	0.1832	0.6001	0.3684	0.060*	
Cl1	0.56577 (14)	1.46719 (8)	0.22217 (6)	0.0567 (3)	
Cl2	0.91696 (13)	1.29605 (10)	0.32170 (6)	0.0563 (3)	
O1W	0.0484 (16)	0.9183 (11)	0.5755 (7)	0.080 (4)*	0.25
H1B	0.0526	0.9507	0.6164	0.120*	0.25
H1A	0.0731	0.8530	0.5831	0.120*	0.25

### Atomic displacement parameters (Å^2^)

**Table:**

	*U*^11^	*U*^22^	*U*^33^	*U*^12^	*U*^13^	*U*^23^
N3	0.045 (2)	0.0354 (18)	0.0334 (19)	0.0009 (15)	0.0035 (15)	0.0016 (15)
C16	0.043 (2)	0.042 (2)	0.035 (2)	−0.004 (2)	0.0025 (19)	0.0000 (19)
N4	0.044 (2)	0.0363 (18)	0.0319 (19)	−0.0018 (15)	0.0035 (15)	0.0015 (15)
C13	0.040 (2)	0.037 (2)	0.032 (2)	0.0002 (18)	0.0032 (18)	0.0035 (18)
C14	0.085 (3)	0.054 (3)	0.028 (2)	−0.017 (3)	−0.001 (2)	0.003 (2)
C15	0.078 (3)	0.046 (3)	0.041 (3)	−0.020 (2)	−0.001 (2)	−0.001 (2)
C10	0.039 (2)	0.039 (2)	0.037 (2)	−0.0005 (18)	0.0054 (18)	0.0047 (18)
Zn1	0.0407 (3)	0.0323 (2)	0.0346 (3)	−0.0028 (2)	0.00384 (19)	−0.0020 (2)
N1	0.0362 (19)	0.036 (2)	0.0378 (19)	−0.0013 (15)	0.0044 (15)	0.0014 (15)
N2	0.0329 (18)	0.0384 (18)	0.044 (2)	−0.0008 (15)	0.0069 (15)	0.0023 (15)
C1	0.035 (2)	0.039 (2)	0.051 (3)	0.0049 (19)	0.011 (2)	0.002 (2)
C2	0.039 (2)	0.038 (2)	0.050 (3)	0.0075 (19)	0.003 (2)	0.005 (2)
C3	0.034 (2)	0.047 (2)	0.054 (3)	0.003 (2)	0.009 (2)	0.003 (2)
C4	0.028 (2)	0.039 (2)	0.040 (2)	−0.0021 (17)	0.0032 (17)	0.0047 (19)
C5	0.046 (2)	0.046 (3)	0.039 (2)	0.001 (2)	0.014 (2)	0.002 (2)
C6	0.045 (2)	0.036 (2)	0.044 (2)	0.0053 (19)	0.011 (2)	−0.0004 (18)
C7	0.033 (2)	0.042 (2)	0.037 (2)	−0.0034 (18)	−0.0002 (18)	0.0035 (18)
C8	0.055 (3)	0.050 (3)	0.043 (2)	−0.005 (2)	0.019 (2)	−0.001 (2)
C9	0.057 (3)	0.038 (2)	0.049 (3)	−0.001 (2)	0.018 (2)	−0.003 (2)
C18	0.070 (3)	0.039 (2)	0.039 (3)	−0.008 (2)	0.003 (2)	0.000 (2)
C17	0.077 (3)	0.046 (3)	0.029 (2)	−0.013 (2)	0.002 (2)	−0.005 (2)
C12	0.065 (3)	0.042 (2)	0.040 (3)	−0.012 (2)	−0.004 (2)	−0.001 (2)
C11	0.062 (3)	0.049 (3)	0.034 (2)	−0.006 (2)	−0.005 (2)	0.010 (2)
Cl1	0.0701 (8)	0.0351 (6)	0.0578 (7)	0.0074 (5)	−0.0061 (6)	−0.0028 (5)
Cl2	0.0412 (6)	0.0771 (8)	0.0463 (6)	0.0007 (6)	−0.0021 (5)	0.0011 (6)

### Geometric parameters (Å, º)

**Table:**

N3—C16	1.319 (5)	N2—C4	1.435 (5)
N3—C18	1.365 (5)	C1—H1	0.9300
N3—Zn1^i^	2.024 (3)	C2—C3	1.338 (5)
C16—N4	1.349 (5)	C2—H2	0.9300
C16—H16	0.9300	C3—H3	0.9300
N4—C17	1.374 (5)	C4—C5	1.370 (5)
N4—C13	1.429 (5)	C4—C9	1.375 (5)
C13—C12	1.373 (5)	C5—C6	1.388 (5)
C13—C14	1.376 (5)	C5—H5	0.9300
C14—C15	1.380 (6)	C6—C7	1.385 (5)
C14—H14	0.9300	C6—H6	0.9300
C15—C10	1.379 (5)	C7—C8	1.391 (5)
C15—H15	0.9300	C8—C9	1.377 (5)
C10—C11	1.381 (5)	C8—H8	0.9300
C10—C7	1.486 (5)	C9—H9	0.9300
Zn1—N1	2.021 (3)	C18—C17	1.349 (5)
Zn1—N3^ii^	2.024 (3)	C18—H18	0.9300
Zn1—Cl2	2.2368 (13)	C17—H17	0.9300
Zn1—Cl1	2.2370 (12)	C12—C11	1.381 (5)
N1—C1	1.326 (4)	C12—H12	0.9300
N1—C2	1.375 (5)	C11—H11	0.9300
N2—C1	1.344 (5)	O1W—H1B	0.8500
N2—C3	1.377 (5)	O1W—H1A	0.8499
			
C16—N3—C18	105.7 (3)	C3—C2—H2	125.1
C16—N3—Zn1^i^	126.7 (3)	N1—C2—H2	125.1
C18—N3—Zn1^i^	127.5 (3)	C2—C3—N2	106.8 (3)
N3—C16—N4	111.6 (3)	C2—C3—H3	126.6
N3—C16—H16	124.2	N2—C3—H3	126.6
N4—C16—H16	124.2	C5—C4—C9	120.7 (4)
C16—N4—C17	106.1 (3)	C5—C4—N2	119.7 (3)
C16—N4—C13	126.3 (3)	C9—C4—N2	119.6 (4)
C17—N4—C13	127.6 (3)	C4—C5—C6	119.9 (4)
C12—C13—C14	119.4 (4)	C4—C5—H5	120.1
C12—C13—N4	121.1 (3)	C6—C5—H5	120.1
C14—C13—N4	119.4 (3)	C7—C6—C5	120.5 (4)
C13—C14—C15	119.7 (4)	C7—C6—H6	119.8
C13—C14—H14	120.1	C5—C6—H6	119.8
C15—C14—H14	120.1	C6—C7—C8	118.3 (4)
C10—C15—C14	122.1 (4)	C6—C7—C10	121.4 (4)
C10—C15—H15	118.9	C8—C7—C10	120.2 (4)
C14—C15—H15	118.9	C9—C8—C7	121.3 (4)
C15—C10—C11	116.9 (4)	C9—C8—H8	119.4
C15—C10—C7	120.3 (4)	C7—C8—H8	119.4
C11—C10—C7	122.8 (3)	C4—C9—C8	119.4 (4)
N1—Zn1—N3^ii^	106.86 (12)	C4—C9—H9	120.3
N1—Zn1—Cl2	105.84 (9)	C8—C9—H9	120.3
N3^ii^—Zn1—Cl2	112.85 (10)	C17—C18—N3	109.7 (4)
N1—Zn1—Cl1	110.21 (9)	C17—C18—H18	125.2
N3^ii^—Zn1—Cl1	106.34 (10)	N3—C18—H18	125.2
Cl2—Zn1—Cl1	114.52 (5)	C18—C17—N4	106.9 (3)
C1—N1—C2	105.6 (3)	C18—C17—H17	126.6
C1—N1—Zn1	127.6 (3)	N4—C17—H17	126.6
C2—N1—Zn1	126.9 (3)	C13—C12—C11	119.9 (4)
C1—N2—C3	106.8 (3)	C13—C12—H12	120.0
C1—N2—C4	127.0 (3)	C11—C12—H12	120.0
C3—N2—C4	126.2 (3)	C12—C11—C10	121.8 (4)
N1—C1—N2	111.1 (3)	C12—C11—H11	119.1
N1—C1—H1	124.5	C10—C11—H11	119.1
N2—C1—H1	124.5	H1B—O1W—H1A	110.4
C3—C2—N1	109.8 (4)		
			
C18—N3—C16—N4	−0.7 (4)	C1—N2—C4—C5	−45.5 (5)
Zn1^i^—N3—C16—N4	−179.6 (2)	C3—N2—C4—C5	132.2 (4)
N3—C16—N4—C17	1.0 (4)	C1—N2—C4—C9	136.4 (4)
N3—C16—N4—C13	178.8 (3)	C3—N2—C4—C9	−46.0 (5)
C16—N4—C13—C12	−159.4 (4)	C9—C4—C5—C6	0.1 (6)
C17—N4—C13—C12	17.9 (6)	N2—C4—C5—C6	−178.0 (3)
C16—N4—C13—C14	20.2 (6)	C4—C5—C6—C7	0.3 (6)
C17—N4—C13—C14	−162.4 (4)	C5—C6—C7—C8	−1.0 (6)
C12—C13—C14—C15	3.1 (7)	C5—C6—C7—C10	179.2 (4)
N4—C13—C14—C15	−176.6 (4)	C15—C10—C7—C6	129.7 (4)
C13—C14—C15—C10	−1.8 (7)	C11—C10—C7—C6	−51.0 (6)
C14—C15—C10—C11	−1.5 (7)	C15—C10—C7—C8	−50.1 (6)
C14—C15—C10—C7	177.8 (4)	C11—C10—C7—C8	129.3 (4)
N3^ii^—Zn1—N1—C1	70.1 (3)	C6—C7—C8—C9	1.3 (6)
Cl2—Zn1—N1—C1	−50.4 (3)	C10—C7—C8—C9	−178.9 (4)
Cl1—Zn1—N1—C1	−174.8 (3)	C5—C4—C9—C8	0.2 (6)
N3^ii^—Zn1—N1—C2	−111.1 (3)	N2—C4—C9—C8	178.4 (4)
Cl2—Zn1—N1—C2	128.4 (3)	C7—C8—C9—C4	−1.0 (6)
Cl1—Zn1—N1—C2	4.0 (3)	C16—N3—C18—C17	0.1 (5)
C2—N1—C1—N2	−0.4 (4)	Zn1^i^—N3—C18—C17	179.0 (3)
Zn1—N1—C1—N2	178.6 (2)	N3—C18—C17—N4	0.5 (5)
C3—N2—C1—N1	0.4 (4)	C16—N4—C17—C18	−0.9 (5)
C4—N2—C1—N1	178.5 (3)	C13—N4—C17—C18	−178.6 (4)
C1—N1—C2—C3	0.2 (4)	C14—C13—C12—C11	−1.2 (6)
Zn1—N1—C2—C3	−178.8 (3)	N4—C13—C12—C11	178.5 (4)
N1—C2—C3—N2	0.1 (5)	C13—C12—C11—C10	−2.3 (7)
C1—N2—C3—C2	−0.3 (4)	C15—C10—C11—C12	3.6 (6)
C4—N2—C3—C2	−178.4 (3)	C7—C10—C11—C12	−175.8 (4)

Symmetry codes: (i) *x*−1/2, −*y*+3/2, *z*+1/2; (ii) *x*+1/2, −*y*+3/2, *z*−1/2.

### Hydrogen-bond geometry (Å, º)

**Table:**

*D*—H···*A*	*D*—H	H···*A*	*D*···*A*	*D*—H···*A*
O1*W*—H1*A*···Cl2^iii^	0.85	2.55	3.270 (14)	143
O1*W*—H1*B*···Cl1^iv^	0.85	2.19	3.038 (14)	179

Symmetry codes: (iii) −*x*+1, −*y*+2, −*z*+1; (iv) *x*−1/2, −*y*+5/2, *z*+1/2.
